# Letter to the editor: Study Summary - Randomized Control Trial of Omega-3 Fatty Acid Supplementation for the Treatment of COVID-19 Related Olfactory Dysfunction

**DOI:** 10.1186/s13063-020-04905-y

**Published:** 2020-11-23

**Authors:** David Lerner, Katherine Garvey, Annie Arrighi-Allisan, Andrey Filimonov, Peter Filip, Katherine Liu, Sen Ninan, Madeleine Schaberg, Patrick Colley, Anthony Del Signore, Satish Govindaraj, Alfred Marc Iloreta

**Affiliations:** Department of Otolaryngology, Icahn School of Medicine at Mount Sinai, New York, NY USA

**Keywords:** COVID-19, Randomised controlled trial, protocol, olfactory dysfunction, omega-3 fatty acid, smell loss

## Abstract

**Objectives:**

To evaluate a therapeutic role for omega-3 fatty acid supplementation in the treatment of olfactory dysfunction associated with COVID-19 infection

**Trial design:**

Randomized, double-blinded, placebo-controlled trial

**Participants:**

Eligible patients are adults with self-reported new-onset olfactory dysfunction of any duration associated with laboratory-confirmed or clinically suspected COVID-19 patients. Exclusion criteria include patients with pre-existing olfactory dysfunction, history of chronic rhinosinusitis or history of sinus surgery, current use of nasal steroid sprays or omega-3 supplementation, fish allergy, or inability to provide informed consent for any reason. The trial is conducted at Mount Sinai Hospital

**Intervention and comparator:**

The intervention group will receive 2000 mg daily of omega-3 supplementation in the form of two “Fish Oil, Ultra Omega-3” capsules (product of Pharmavite®) daily. The comparator group will take 2 placebo capsules of identical size, shape, and odor daily for 6 weeks.

**Main outcomes:**

Each subject will take a Brief Smell Identification Test at study enrolment and completion after 6 weeks. The primary outcome will be change in Brief Smell Identification Test over the 6-week period.

**Randomisation:**

Patients will be randomized by the Investigational Drug Pharmacy at the Icahn School of Medicine at Sinai via a computer-generated sequence in a 1:1 allocation to treatment or control arms.

**Blinding (masking):**

Both participants and researchers will be blinded.

**Numbers to be randomised (sample size):**

There will be 88 participants randomized to each group. A total of 176 participants will be randomized.

**Trial Status:**

Protocol Version 1, 8/3/2020

Recruitment is ongoing, started 8/5/2020 with estimated completion 11/30/2020.

**Trial registration:**

The trial is registered on ClinicalTrials.gov with Protocol Identifier: NCT04495816.

Trial registration: ClinicalTrials.gov, NCT04495816. Registered 3 August 2020

**Full protocol:**

The full protocol is attached as an additional file, accessible from the Trials website (Additional file 1).

## Supplementary Information

**Additional file 1.** Full Study Protocol.

## Data Availability

The final trial dataset will be accessible from the author on reasonable request. Contact David Lerner (e-mail david.lerner2@mountsinai.org).

