# Supplementary material for: Letter to the editor: Study Summary - Randomized Control Trial of Omega-3 Fatty Acid Supplementation for the Treatment of COVID-19 Related Olfactory Dysfunction
Source: Trials. 2020 Nov 23;21:942. doi: 10.1186/s13063-020-04905-y (PMC7681177; doi:10.1186/s13063-020-04905-y)
Supplement: Supplementary file 1 — Additional file 1. Full Study Protocol. [file 13063_2020_4905_MOESM1_ESM.docx]

**Omega-3 Supplementation for the Treatment of COVID-19 Infection-Related Olfactory Dysfunction**

David Lerner MD^1^, Katherine Garvey BA^1^, Annie Arrighi-Allisan^1^, Andrey Filimonov MD^1^, Peter Filip MD^1^, Katherine Liu BA^1^, Sen Ninan BA, Madeleine Schaberg MD, Patrick Colley MD, Anthony Del Signore MD, Satish Govindaraj MD, Alfred Marc Iloreta M

^1^Department of Otolaryngology, Icahn School of Medicine at Mount Sinai, New York, NY, USA

**Abstract**

**Objectives**

To evaluate a therapeutic role for omega-3 fatty acid supplementation in the treatment of olfactory dysfunction associated with COVID-19 infection

**Trial design**

Randomized, double-blinded, placebo-controlled trial

**Participants**

Eligible patients are adults with self-reported new-onset olfactory dysfunction of any duration associated with laboratory-confirmed or clinically suspected COVID-19 patients. Exclusion criteria include patients with pre-existing olfactory dysfunction, history of chronic rhinosinusitis or history of sinus surgery, current use of nasal steroid sprays or omega-3 supplementation, fish allergy, or inability to provide informed consent for any reason. The trial is conducted at Mount Sinai Hospital

**Intervention and comparator**

The intervention group will receive 2000 mg daily of omega-3 supplementation in the form of two “Fish Oil, Ultra Omega-3” capsules (product of Pharmavite®) daily. The comparator group will take 2 placebo capsules of identical size, shape, and odor daily for 6 weeks.

**Main outcomes**

Each subject will take a Brief Smell Identification Test at study enrolment and completion after 6 weeks. The primary outcome will be change in Brief Smell Identification Test over the 6-week period.

**Randomisation**

Patients will be randomized by the Investigational Drug Pharmacy at the Icahn School of Medicine at Sinai via a computer-generated sequence in a 1:1 allocation to treatment or control arms.

**Blinding (masking)**

Both participants and researchers will be blinded.

**Numbers to be randomised (sample size)**

A total of 176 participants will be randomized. There will be 88 participants randomized to each group.

**Trial Status**

Protocol Version 1, 8/3/2020

Recruitment is ongoing, started 8/5/2020 with estimated completion 11/30/2020.
**Trial registration**

The trial is registered on ClinicalTrials.gov with Protocol Identifier: NCT04495816.

Trial registration: ClinicalTrials.gov, NCT04495816. Registered 3 Aug 2020 https://clinicaltrials.gov/ct2/show/NCT04495816

**Background and Significance:**

Infection with the severe acute respiratory syndrome coronavirus 2 (SARS-CoV-2) has been linked to new-onset olfactory dysfunction, often as the only presenting symptom.^1-5^ Reports of hyposmia or anosmia among patients with confirmed SARS-CoV-2 infection vary widely among the literature, with one meta-analysis including 1627 patients reporting a prevalence of 43.9%.^4,6,7^ Patients with olfactory dysfunction following SARS-CoV-2 infection often demonstrate improvement 1-2 weeks after symptom onset, but despite promising reports of early recovery in short-term follow-up studies a substantial portion of patients continue to display partial or complete smell loss.^8,9^

There is no consensus on pathogenesis of SARS-CoV-2-associated olfactory dysfunction, although many have suggested that the virus gains access to the olfactory system through a variety of potential cellular targets expressing angiotensin-converting enzyme 2 (ACE-2) within the nasal passageway, including olfactory epithelium or olfactory support cells.^5,10^ Animal studies of olfactory dysfunction after common Coronavirus infections found decreased olfactory neuronal lifespan despite minimal neuroepithelial damage, suggesting a mechanism at the level of the olfactory bulb.^11-13^

Anosmia most commonly arises in association with sinonasal disease or post-infectious or post-traumatic disorders.^14,15^ Notably, olfactory loss has been associated with impaired quality of life, higher rates of depression, and even increased mortality risk.^14,16,17^ Spontaneous recovery has been observed in patients with post-infectious olfactory dysfunction, typically over a period of months to years, with an estimated one-third of patients demonstrating meaningful improvement after one year.^18-21^

Smell retraining therapy may be an effective therapeutic option for patients with post-infectious olfactory dysfunction, particularly for patients who initiate treatment within one year from onset of symptoms.^14,22,23^ Despite improvement with smell retraining therapy, it requires an intervention period usually of at least 6 months and patients with anosmia are not typically expected to have full recovery.^24^ Various pharmacotherapies have been investigated in the treatment of post-infectious anosmia but none have clearly demonstrated utility with the exception of a possible benefit for nasal steroid irrigations in combination with smell retraining therapy.^25-27^

Omega-3 polyunsaturated fatty acid supplementation is a potential intervention for olfactory dysfunction in patients without sinonasal disease. Inflammatory processes are known to contribute to a range of diseases, which has caused increased attention on a potential therapeutic role of omega-3 fatty acids. Eicosapentaenoic acid (EPA) and docosahexaenoic acid (DHA) are omega-3 fatty acids commonly found in fish and fish oil supplements that are known to inhibit a variety of cellular inflammatory pathways including the production of pro-inflammatory cytokines.^28^

Animal studies have shown that omega-3 fatty-acid deficiency in mice is associated with olfactory dysfunction at baseline^29^ and omega-3 fatty acid supplementation in mice following spinal cord injury leads to increased levels of anti-inflammatory and anti-oxidant metabolic activity within spinal cord cells.^30^ Additionally, following peripheral nerve injury mice with higher levels of endogenous omega-3 fatty acids have demonstrated improved recovery from peripheral nerve injury.^31^ In humans, a large cross-sectional study found that older adults with higher dietary fat intake had lower incidence of olfactory impairment.^32^ From a clinical perspective, patients without sinonasal disease receiving post-operative omega-3 fatty acid supplementation after endoscopic endonasal skull base surgery in a randomized control trial demonstrated a significantly greater rate of return of normal olfactory dysfunction.^33^

There are many unanswered questions regarding the natural history of SARS-CoV-2-associated olfactory dysfunction, as well as potential therapeutic interventions. We hope to gain a better understanding of each through a randomized double-blind placebo control study that assesses both objective and subjective olfactory dysfunction.

**Setting and Subject Eligibility:**

This study will be executed at the Icahn School of Medicine at Mount Sinai. Patients will be recruited from the practices of faculty in the Department of Otolaryngology – Head and Neck Surgery and via the STOP COVID web-based application. All research will be conducted at the Icahn School of Medicine at Mount Sinai.Each subject will be screened via telephone call for inclusion using our inclusion and exclusion criteria. Patients who qualify will then be sent a Brief Smell Identification Test (BSIT) via mail. Subjects who have objective evidence of hyposmia, defined in our study as a score of 8 or fewer correctly answered questions on our 12-question survey, will proceed in our study.

Eligibility will be based on the inclusion and exclusion criteria below:

Inclusion Criteria

-Adults (≥18 years of age)

-Self-reported new-onset olfactory dysfunction of any duration

-Laboratory-confirmed or clinically suspected COVID-19 infection

Exclusion Criteria

-Patients <18 years of age

-Patients who are unable to provide informed consent

-Patients with pre-existing self-reported olfactory dysfunction

-Patients with a history of chronic nasal/sinus infections (rhinosinusitis) or history of endoscopic sinus surgery

-Patients using nasal steroid sprays or irrigations for any reason

-Patients who are prisoners of the state

-Patients who have psychiatric or developmental disorder conditions that may impair ability to provide informed consent

-Patients with allergy to fish oil or omega-3 supplementation

-Patients who do not consume fish for any reason

**Study Design**

Eligible patients will be randomized by the Mount Sinai Investigational Drug Pharmacy via a computer-generated sequence in a 1:1 allocation to treatment or control arms. Researchers and subjects will remind blinded to patients’ experimental group throughout the duration of the study. Subjects in the control group will take 2 capsule daily of “1400mg Fish Oil, Ultra Omega-3, Burp-Less” provided by Pharmavite ® for a total of 2000 mg omega-3 fatty acids daily for 6 weeks. Patients randomized to the control group will receive a placebo produced by the manufacturer to be of identical size, shape, and, taste as the active capsules. Patients receiving placebo will follow the same regimen as the treatment group. Collection of data will occur through the use of the electronic medical record system, through phone conversations between investigators and subjects, and through information collected during office visits.

The primary outcome studied will be the BSIT, which will be administered 6 weeks of study participation. The secondary outcomes include patient-reported outcome measure surveys, a modified Brief Questionnaire of Olfactory Dysfunction - Negative Statements (QOD-NS) survey and Sino-Nasal Outcome Test (SNOT-22), administered at time points of 1, 2, 4, and 6 weeks after symptom onset. The Brief Smell Identification Test will be sent via mail while the self-reported questionnaires will be sent over email, with follow-up telephone calls for patients who do not respond to email requests.

The primary endpoint is change in 6-week BSIT scores from baseline. Secondary endpoints include QOD-NS and SNOT-22 scores. Non-parametric Mann Whitney U test will be used to compare experimental and control groups, assuming that outcomes will not have a normal distribution.

**Declarations**

**Ethics approval and consent to participate**

This study protocol was approved by the Institutional Review Board at the Icahn School of Medicine at Mount Sinai on 7/10/2020 (HS#:20-00511, GCO#20-1132 ISMMS).

Informed consent will be obtained from all participants. Only adults are included in the study.

**Consent for publication**

Not applicable

**Availability of data and materials**

The final trial dataset will be accessible from the author on reasonable request. Contact David Lerner (e-mail david.lerner2@mountsinai.org).

**Competing interests**

The authors declare that they have no competing interests.

**Funding**

No funding was received for this study. Study drug and capsules were provided by Pharmavite®. Pharmavite® had no role in the design of the study and collection, analysis and interpretation of data and in the writing of the manuscript.

**Authors' contributions**

DL, AF, PF – study design, subject recruitment

KG, AA, KL, SN – subject recruitment, literature review

MS, PC, AS, SG – study design, subject recruitment

AI – study design, study oversight

**References**

1. Vaira LA, Salzano G, Deiana G, De Riu G. Anosmia and ageusia: common findings in COVID-19 patients. *Laryngoscope.* 2020.

2. Eliezer M, Hautefort C, Hamel AL, et al. Sudden and Complete Olfactory Loss Function as a Possible Symptom of COVID-19. *JAMA Otolaryngol Head Neck Surg.* 2020.

3. Vukkadala N, Qian ZJ, Holsinger FC, Patel ZM, Rosenthal E. COVID-19 and the otolaryngologist - preliminary evidence-based review. *Laryngoscope.* 2020.

4. Moein ST, Hashemian SM, Mansourafshar B, Khorram-Tousi A, Tabarsi P, Doty RL. Smell dysfunction: a biomarker for COVID-19. *Int Forum Allergy Rhinol.* 2020.

5. Kaye R, Chang CWD, Kazahaya K, Brereton J, Denneny JC, 3rd. COVID-19 Anosmia Reporting Tool: Initial Findings. *Otolaryngol Head Neck Surg.* 2020;163(1):132-134.

6. Tong JY, Wong A, Zhu D, Fastenberg JH, Tham T. The Prevalence of Olfactory and Gustatory Dysfunction in COVID-19 Patients: A Systematic Review and Meta-analysis. *Otolaryngol Head Neck Surg.* 2020;163(1):3-11.

7. Lechien JR, Chiesa-Estomba CM, De Siati DR, et al. Olfactory and gustatory dysfunctions as a clinical presentation of mild-to-moderate forms of the coronavirus disease (COVID-19): a multicenter European study. *Eur Arch Otorhinolaryngol.* 2020.

8. Chiesa-Estomba CM, Lechien JR, Radulesco T, et al. Patterns of smell recovery in 751 patients affected by the COVID-19 outbreak. *Eur J Neurol.* 2020.

9. Reiter ER, Coelho DH, Kons ZA, Costanzo RM. Subjective smell and taste changes during the COVID-19 pandemic: Short term recovery. *Am J Otolaryngol.* 2020;41(6):102639.

10. Vaira LA, Salzano G, Fois AG, Piombino P, De Riu G. Potential pathogenesis of ageusia and anosmia in COVID-19 patients. *Int Forum Allergy Rhinol.* 2020.

11. Netland J, Meyerholz DK, Moore S, Cassell M, Perlman S. Severe acute respiratory syndrome coronavirus infection causes neuronal death in the absence of encephalitis in mice transgenic for human ACE2. *J Virol.* 2008;82(15):7264-7275.

12. Youngentob SL, Schwob JE, Saha S, Manglapus G, Jubelt B. Functional consequences following infection of the olfactory system by intranasal infusion of the olfactory bulb line variant (OBLV) of mouse hepatitis strain JHM. *Chem Senses.* 2001;26(8):953-963.

13. Schwob JE, Saha S, Youngentob SL, Jubelt B. Intranasal inoculation with the olfactory bulb line variant of mouse hepatitis virus causes extensive destruction of the olfactory bulb and accelerated turnover of neurons in the olfactory epithelium of mice. *Chem Senses.* 2001;26(8):937-952.

14. Boesveldt S, Postma EM, Boak D, et al. Anosmia-A Clinical Review. *Chem Senses.* 2017;42(7):513-523.

15. Fonteyn S, Huart C, Deggouj N, Collet S, Eloy P, Rombaux P. Non-sinonasal-related olfactory dysfunction: A cohort of 496 patients. *Eur Ann Otorhinolaryngol Head Neck Dis.* 2014;131(2):87-91.

16. Croy I, Nordin S, Hummel T. Olfactory disorders and quality of life--an updated review. *Chem Senses.* 2014;39(3):185-194.

17. Ekstrom I, Sjolund S, Nordin S, et al. Smell Loss Predicts Mortality Risk Regardless of Dementia Conversion. *J Am Geriatr Soc.* 2017;65(6):1238-1243.

18. Cavazzana A, Larsson M, Munch M, Hahner A, Hummel T. Postinfectious olfactory loss: A retrospective study on 791 patients. *Laryngoscope.* 2018;128(1):10-15.

19. Reden J, Mueller A, Mueller C, et al. Recovery of olfactory function following closed head injury or infections of the upper respiratory tract. *Arch Otolaryngol Head Neck Surg.* 2006;132(3):265-269.

20. Hendriks AP. Olfactory dysfunction. *Rhinology.* 1988;26(4):229-251.

21. Reden J, Herting B, Lill K, Kern R, Hummel T. Treatment of postinfectious olfactory disorders with minocycline: a double-blind, placebo-controlled study. *Laryngoscope.* 2011;121(3):679-682.

22. Sorokowska A, Drechsler E, Karwowski M, Hummel T. Effects of olfactory training: a meta-analysis. *Rhinology.* 2017;55(1):17-26.

23. Pekala K, Chandra RK, Turner JH. Efficacy of olfactory training in patients with olfactory loss: a systematic review and meta-analysis. *Int Forum Allergy Rhinol.* 2016;6(3):299-307.

24. Soler ZM, Patel ZM, Turner JH, Holbrook EH. A primer on viral-associated olfactory loss in the era of COVID-19. *Int Forum Allergy Rhinol.* 2020;10(7):814-820.

25. Damm M, Pikart LK, Reimann H, et al. Olfactory training is helpful in postinfectious olfactory loss: a randomized, controlled, multicenter study. *Laryngoscope.* 2014;124(4):826-831.

26. Yan CH, Overdevest JB, Patel ZM. Therapeutic use of steroids in non-chronic rhinosinusitis olfactory dysfunction: a systematic evidence-based review with recommendations. *Int Forum Allergy Rhinol.* 2019;9(2):165-176.

27. Nguyen TP, Patel ZM. Budesonide irrigation with olfactory training improves outcomes compared with olfactory training alone in patients with olfactory loss. *Int Forum Allergy Rhinol.* 2018;8(9):977-981.

28. Calder PC. Omega-3 fatty acids and inflammatory processes: from molecules to man. *Biochem Soc Trans.* 2017;45(5):1105-1115.

29. Greiner RS, Moriguchi T, Slotnick BM, Hutton A, Salem N. Olfactory discrimination deficits in n-3 fatty acid-deficient rats. *Physiol Behav.* 2001;72(3):379-385.

30. Figueroa JD, De Leon M. Neurorestorative targets of dietary long-chain omega-3 fatty acids in neurological injury. *Mol Neurobiol.* 2014;50(1):197-213.

31. Gladman SJ, Huang W, Lim SN, et al. Improved outcome after peripheral nerve injury in mice with increased levels of endogenous omega-3 polyunsaturated fatty acids. *J Neurosci.* 2012;32(2):563-571.

32. Gopinath B, Sue CM, Flood VM, Burlutsky G, Mitchell P. Dietary intakes of fats, fish and nuts and olfactory impairment in older adults. *Br J Nutr.* 2015;114(2):240-247.

33. Yan CH, Rathor A, Krook K, et al. Effect of Omega-3 Supplementation in Patients With Smell Dysfunction Following Endoscopic Sellar and Parasellar Tumor Resection: A Multicenter Prospective Randomized Controlled Trial. *Neurosurgery.* 2020.
